# Stimulus- and Neural-Referred Visual Receptive Field Properties following Hemispherectomy: A Case Study Revisited

**DOI:** 10.1155/2019/6067871

**Published:** 2019-09-03

**Authors:** Hinke N. Halbertsma, Koen V. Haak, Frans W. Cornelissen

**Affiliations:** ^1^Laboratory of Experimental Ophthalmology-Visual Neurosciences, University Medical Center Groningen, 9713 GZ Groningen, Netherlands; ^2^Donders Institute of Brain, Cognition and Behavior, Radboud University Medical Center, 6525 GA Nijmegen, Netherlands

## Abstract

Damage to the visual system can result in (a partial) loss of vision, in response to which the visual system may functionally reorganize. Yet the timing, extent, and conditions under which this occurs are not well understood. Hence, studies in individuals with diverse congenital and acquired conditions and using various methods are needed to better understand this. In the present study, we examined the visual system of a young girl who received a hemispherectomy at the age of three and who consequently suffered from hemianopia. We did so by evaluating the corticocortical and retinocortical projections in the visual system of her remaining hemisphere. For the examination of these aspects, we analyzed the characteristics of the connective fields (“neural-referred” receptive fields) based on both resting-state (RS) and retinotopy data. The evaluation of RS data, reflecting brain activity independent from visual stimulation, is of particular interest as it is not biased by the patient's atypical visual percept. We found that, primarily when the patient was at rest, the connective fields between V1 and both early and late visual areas were larger than normal. These abnormally large connective fields could be a sign either of functional reorganization or of unmasked suppressive feedback signals that are normally masked by interhemispheric signals. Furthermore, we confirmed our previous finding of abnormal retinocortical or “stimulus-referred” projections in both early and late visual areas. More specifically, we found an enlarged foveal representation and smaller population receptive fields. These differences could also be a sign of functional reorganization or rather a reflection of the interruption visual information that travels, via the remainder of the visual pathway, from the retina to the visual cortex. To conclude, while we do find indications for relatively subtle changes in visual field map properties, we found no evidence of large-scale reorganization—even though the patient could have benefitted from this. Our work suggests that at a later developmental stage, large-scale reorganization of the visual system no longer occurs, while small-scale properties may still change to facilitate adaptive processing and viewing strategies.

## 1. Introduction

Damage to critical components of its circuitry can have major consequences for the visual system. For example, lesions at the level of the optic radiation or early visual cortex can result in visual field defects spanning part of, or the entire, hemifield. Evidence supporting a reorganizatory potential of the visual system following both early and late acquired brain damage is now emerging (see for reviews [1–6]). For rehabilitation purposes, it is critical to understand the degree to which this potential can be deployed to restore lost function. For this reason, detailed studies on the organization of the visual system in patients and healthy observers are essential.

In the present study, we revisited the case of a girl who, at the age of three, received hemispherectomy as a treatment for Rasmussen syndrome (chronic focal encephalitis) and intractable epilepsy [7]. Her left hemisphere had been removed completely, which resulted in a full right homonymous visual field defect. The acquired visual field defect allowed us to examine the potential reorganization of functional regions through examination of the properties of the visual field maps in her remaining hemisphere. Specifically, it allowed us to examine the effects of an interrupted processing of half of the retinal output (i.e., visual information coming from the left visual field) and the deprivation of interhemispheric inputs on the visual field map (VFM) properties.

We originally examined the VFM using retinotopy based on functional magnetic resonance imaging (fMRI) and established an absence of large-scale reorganization. However, using a specific analysis technique called population receptive field (pRF) modeling, we were able to assess more detailed properties of the underlying neuronal architecture. These pRF properties describe how the visual field is neuronally represented in the visual cortex. Because pRFs are modeled based on signals acquired during active stimulation, they are also referred to as “stimulus-referred” receptive fields. The application of pRF modelling to this patient suggested the presence of abnormal pRF properties in the visual areas [7]. Due to methodological developments since that study, we now had the opportunity to investigate this patient more extensively using connective field (CF) modeling. CF modeling, as an extension of the pRF technique, assesses the intracortical receptive fields, which are therefore also called “neural-referred” receptive fields [8]. The properties of the CFs describe how the visual field representations are projected from one region in the brain to another. A particular interesting aspect of CFs and the resulting CF maps is that we can estimate these, in addition to “active” stimulus-driven data, also based on resting-state data [9]. This has the advantage that the underlying signals are based on spontaneous fluctuations in brain activity and thus do not rely on visual input, which in neurological or ophthalmic patients may already be altered due to changes at the level of the eye or elsewhere in the brain [10].

In addition to the detailed evaluation of the CF maps, we also revisited pRF mapping (based on the “active” stimulus-driven data). Where our previous work focused on pRF modeling using a single unilateral pRF model, we additionally applied bilateral pRF modelling to examine a potential ipsilateral contribution to the patient's cortical VFMs. Ipsilateral VFM representations have been reported in cases with a congenital absence of or acquired damage to one of the two cerebral hemispheres [11–13]. In the interest of replication, and as a further improvement on our previous report, we also reevaluated the unilateral pRF model parameters of the patient. Yet, in addition to examining only differences in average or median values, we also compared parameter distributions between the patient and controls to assess whether changes occurred in specific parameter ranges.

## 2. Evaluation of Visual Field Map Properties

### 2.1. Materials and Methods

#### 2.1.1. Participants

**Figure 1:**
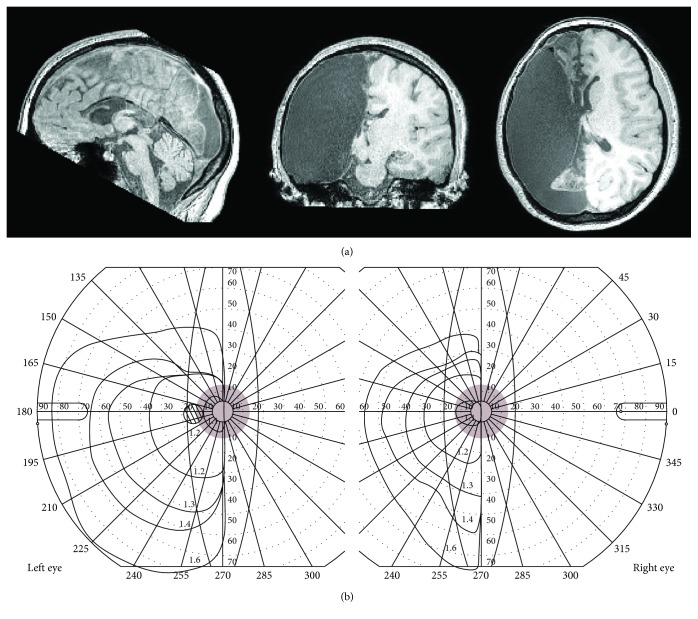
Anatomical MRI and Goldmann perimetry of the hemispherectomised patient. (a) High-resolution anatomical MRI scan showing the absence of the left hemispheres from a sagittal (left), coronal (middle), and axial (right) view. (b) Goldmann perimetry of both the left and right eyes showing a complete right-sided homonymous visual field defect, without macular sparing. The red patch indicates the size of the visual field that was stimulated during the fMRI experiment.

In this case-control study, the VFM properties of the right hemisphere of a 16-year-old female hemispherectomy patient were investigated. At the age of three, the girl's left hemisphere was removed completely (see Figure 1(a) for an anatomical MRI image of her brain). This surgical procedure resulted in a full right homonymous visual field defect without macular sparing (see Figure 1(b) for the results of a Goldmann perimetry). In spite of her visual defect, the girl was able to fixate reliably (as can also been seen from the straight visual field boundary along the vertical meridian of the Goldmann perimetry). At the same time, despite an initial major impact on her motoric and linguistic abilities, the girl partially recovered her motor control and speaks bilingually.

Twelve young healthy female participants (mean age = 22, sd = 1.8 years), of whom data was already acquired for a different project [14], with normal or corrected-to-normal visual acuity served as a control group. An additional four young healthy female participants (mean age = 27.5, sd = 1.7 years) with normal or corrected-to-normal visual acuity were recruited. This additional cohort served to control for the effect of different viewing instructions during RS on the estimation of the CF properties (see last paragraph of Data Acquisition).

For each participant, a high-resolution structural magnetic resonance (MR) image and a series of functional MR images were made available or newly collected. Informed written consent was obtained from all observers in accordance with the study procedures and protocols approved by the Medical Ethical Committee of the University Medical Center Groningen, the Netherlands. As the patient and the control participants were scanned at different points in time and initially for two different project, there were slight differences in the MR acquisition and visual stimulation protocols.

#### 2.1.2. Stimulus

Visual field maps (VFM) were localized using a retinotopic mapping paradigm. The stimulus used was a drifting bar with a high-contrast checkerboard pattern and was presented on a grey (mean luminance) background. The bar had a radius of 12 or 10.21 degrees of visual angle and width of 3 and 2.75 degrees of visual angle, for the patient and controls, respectively. The full stimulus presentation (192 seconds) consisted of a sequence of eight bar apertures with four different orientations and two opposite motion directions (motion step every 1.5 seconds). Four 12-second mean-luminance periods (“blank periods”) were inserted that replaced the bar presentation when it traversed the visual field along the diagonals. To ensure participants' central fixation, a small dot was presented in the center of the screen. Participants were asked to press a button on a button box whenever the dot changed color. The full stimulus cycle was presented to the participants either four or six times, for the patient and the controls, respectively, during separate scans. The VISTADISP toolbox (VISTA Lab, Stanford University) and PsychToolbox (https://github.com/Psychtoolbox-3/Psychtoolbox-3/) were used for stimulus control and display. For the patient, the stimulus was back-projected on a translucent display (44 × 34 cm) using a Barco LCD projector G300 set at a resolution of 800 × 600 pixels. For the control participants, the stimulus was presented on a 24-inch BOLD screen, an MRI-compatible full-color H-IPS LCD, and set at a resolution of 1920 × 1200 pixels. All participants viewed the display through a mirror placed at 11 cm from the eyes, with a viewing distance of 64 cm and 118 cm, for the patient and the controls, respectively.

#### 2.1.3. Data Acquisition

All MRI images were acquired, using a 32-channel head coil, using a 3T Philips Intera MRI scanner (Philips; Best, the Netherlands) at the Neuroimaging Center in Groningen, the Netherlands.

For the patient, six functional scans (i.e., four retinotopy (RET) and two resting-state (RS) scans) were obtained using echo-planar imaging (EPI; TR_ret_ = 1500 ms/TR_rs_ = 2000 ms, FOV_ret_ = 194 × 72 × 244 mm/FOV_rs_ = 192 × 144 × 192 mm, voxel − size_ret_ = 2.33 × 2.33 × 3 mm/voxel − size_rs_ = 3 × 3 × 3 mm). During the RS scans, the patient was instructed to keep her eyes open and do nothing. Each RS scan had a duration of 370 seconds during which 181 volumes of each 48 slices were obtained. Both RS scans were obtained after the four RET scans. During retinotopy, the patient was instructed to pay attention to the stimulus and maintain fixation. In principle, each RET scan had a duration of 204 seconds (including a prescan period of twelve seconds), during which 136 volumes of each 24 slices were obtained. For some RET scans, the scanner was on longer than the actual experiment duration lasted; hence, additional acquired volumes were discarded.

For every control participant, seven EPI scans (i.e., one RS scan and six RET scans) were obtained or were made available (TR_rs_ = 2000 ms/TR_ret_ = 1500 ms, FOV_rs_ = 220 × 121 × 220 mm/FOV_ret_ = 224 × 72 × 193.5 mm, voxel − size_rs_ = 3.44 × 3.44 × 3.29 mm/voxel − size_ret_ = 2.33 × 2.33 × 3 mm). During the RS scan, control participants were instructed to keep their eyes closed and do nothing. The RS scan had a duration of 708 seconds, during which 350 volumes of each 37 slices were obtained. During retinotopy, the control participants were instructed to pay attention to the stimulus and maintain fixation. Each RET scan had a duration of 204 seconds (including a prescan period of twelve seconds), during which 136 volumes of each 24 slices were obtained. However, due to technical issues, some scans lasted for only 198 seconds. As a consequence, no BOLD responses to the last two frames of the stimulus presentation (corresponding to a blank period) were recorded. A correction for this has been implemented in the pRF model.

The cohort of twelve control participants was initially scanned with a different project in mind [14]. As a result, the patient and the control subjects had received different instructions during the acquisition of the RS scans. Even though the scanner room was dimmed, the patient was instructed to fixate yet allowed to keep her eyes open. However, no eye tracking was performed leaving some room for fixation instability as a factor, and there was no control on whether she had in fact kept her eyes open during the entire RS scans. The participants that now serve as controls were instructed to keep their eyes closed. It cannot be excluded that these differences in instruction or their execution may have influenced the CF estimates. To verify this, in four additional female control participants, three six-minute EPI RS scans were collected during which participants were instructed, in successive scans, to keep their eyes closed (EC), fixated on a centered cross (FIX), or open (EO). The scanning parameters for these scans were identical to those used for the original control group. Furthermore, in this group of controls, six RET scans were also collected following the same scanning protocol and procedure as the other control participants in order to localize the visual areas.

For each participant, a T1-weighted high-resolution three-dimensional scan was obtained (TR = 9.00 ms, TE = 3.5 ms, flip angle = 8°, acquisition matrix = 251 × 251 × 170 mm, FOV = 256 × 170 × 232, voxel − size = 1 × 1 × 1 mm) over a duration of 251 seconds, with a maximum number of 170 slices. Additionally, a short T1-weighted anatomical scan, with the same inplane resolution and orientation as the RET scans, was acquired for registration purposes of the retinotopy data.

#### 2.1.4. Data Preprocessing

The T1-weighted anatomical scans were reoriented to AC-PC space and subsequently automatically segmented into grey and white matter using FreeSurfer (http://surfer.nmr.mgh.harvard.edu/), the results of which were manually refined using ITK-SNAP 3.6.0 (http://www.itksnap.org). To obtain a cortical reconstruction of the patient's brain, the intact hemisphere was copied and flipped along the *x*-axis to mimic a normal brain. Furthermore, because the tissue contrast in the patient's anatomical scan was suboptimal, we used a white matter (WM) mask created by FSL (https://fsl.fmrib.ox.ac.uk/fsl/fslwiki) FAST to decrease the intensity values for non-WM tissue, which significantly improved the automated cortical reconstruction done by FreeSurfer. All functional data were then preprocessed and analyzed at the individual level using FSL and the mrVista toolbox (https://github.com/vistalab/vistasoft) in MATLAB 2012b. RS data were first corrected for motion using FSL MCFLIRT and then denoised using ICA AROMA [15], a tool for head-motion-related artefact identification and removal. Using mrVista, RS data were interpolated to 1 mm structural data. The time series of the two, six-minute, consecutively collected RS scans of the patient were first normalized over time and then concatenated. This created a time series that matched the length of the RS time series of the controls.

RET data were preprocessed in mrVista. As the RET scans included a prescan of 12 seconds, the first eight volumes were removed from further analysis. Motion correction was applied to every scan; head movements within and between scans were calculated and corrected for (using rigid-body motion compensation [16]). Scans containing sudden head displacements larger than 1.5 voxels were discarded from further analyses. Functional scans were then averaged, interpolated to 1 mm structural data, and projected onto a mesh representation of the cortical surface.

#### 2.1.5. Population Receptive Field Mapping (pRF Mapping)

Population receptive fields (pRFs) were estimated using a model-based analysis (i.e., population receptive field mapping), by fitting 2D Gaussian pRFs to the data, as described in [17]. Parameters of interest were pRF eccentricity, polar angle, and size, where a voxel's pRF size reflects the size of the region of visual space that the voxel responds to and polar angle and eccentricity represent that voxel's pRF center location. The best-fitting models with a variance explained of at least 10% were then projected on an inflated representation of the visual cortex, on which early visual areas (V1, V2, and V3) and late visual areas (V3A, hV4, LO1, and LO2) were delineated based on their retinotopic properties (i.e., polar angle and eccentricity) using standard criteria [17–20]. More specifically, boundaries of the VFMs were based on phase reversals (i.e., reversals in polar angle value) and a maximum eccentricity of 10.2 degrees of visual angle. Furthermore, from every voxel within each ROI, its estimated pRF eccentricity and size were extracted for further analyses (see Section 2.1.8).

#### 2.1.6. Connective Field Mapping (CF Mapping)

Connective fields (CF) measure interareal spatial integration (as reflected by measures of corticocortical correlations) and were estimated using a model-based analysis (i.e., connective field mapping), as described by [8]. Parameters of interest derived were CF size and eccentricity. CF size reflects the size of the cortical surface area within a source region from which a target region samples its information. CF eccentricity equals the pRF eccentricity corresponding to the center of the sampled cortical area. Hence, the smaller the radius of the sampled area of the source region (i.e., the CF size), the higher the sampling resolution from cortical space. Using this CF-mapping approach, the functional connectivity profiles between the different VFMs were charted.

Six “source > target” CF maps (representing either CF eccentricity or size estimations) were computed with V1 as the source ROI and V2, V3, V3A, hV4, LO1, and LO2 as the six target ROIs (tROIs). These CF maps were estimated (separately) from both signals under visual stimulation and spontaneous (resting-state) BOLD signals. For each participant, this resulted in one “active-state” CF (CF_AS_) map and one “resting-state” CF (CF_RS_) map per tROI. From every voxel within the tROIs, its estimated CF eccentricity and size were extracted for further analysis.

Similarly, for the additional four control participants, the CF field parameters were estimated per tROI and for the three viewing conditions (i.e., eyes closed, fixated, and open) separately. This resulted in three CF maps per tROI: CF_EO_, CF_FIX_, and CF_EC_. Again, for each tROI, the estimated CF eccentricity and size were extracted for further analysis.

#### 2.1.7. Bilateral pRF Mapping

In order to test whether each cortical location processes information from a single region of the visual field (as assumed with the conventional one Gaussian pRF model mentioned above) or from two bilateral regions of the visual field, two additional pRF models were run. With these bilateral pRF models, the time series predictions based on two, rather than one single, 2D Gaussians are fitted to the data. The Gaussians were mirrored around either the horizontal meridian (second fit in the ipsilateral hemifield) or the vertical meridian (second fit in the contralateral hemifield). This allowed us to examine the additional value of the second fit to the model prediction (see also [21]). Note that due to the mirroring, the unilateral and both bilateral models have the same number of parameters and can therefore be directly compared in terms of the explained variance in the fMRI time series.

#### 2.1.8. Data Analyses

The derived VFM parameter estimates (i.e., CF size and eccentricity and pRF size and eccentricity) were thresholded at a variance explained (VE) level of 20%. Furthermore, we restricted our analyses to the VFM parameters corresponding to the stimulated region of the visual field of the controls. Hence, CFs and pRFs with eccentricity values larger than 10.2 degrees of visual angle were excluded from further analysis. Regions of interest were grouped into early (i.e., V1, V2, and V3) and late (i.e., V3A, LO1, LO2, and hV4) visual areas, and the CF and pRF parameter estimates were aggregated accordingly. Subsequent analyses were performed on the grouped areas, representing either early or late visual areas.

To examine whether the VFM parameter estimates differed between the patient and the control participants, several comparisons were performed. First, per area group, participants' CF and pRF sizes were plotted against the corresponding eccentricity estimates to examine the relationship between the two parameters. Per bin of 1 degree of CF_AS_, CF_RS_, and pRF eccentricity, the corresponding median sizes were calculated. Linear fits were computed for the median size vs. eccentricity relationship. Furthermore, the 95% confidence intervals (CIs) of the median were computed using a bootstrapping procedure with 1000 iterations.

Second, as a summarizing descriptive, participants' median CF and pRF eccentricities and sizes were computed per area group. Difference scores for each of the parameter's median were calculated for each possible “patient minus control” comparison. Obtained difference scores were statistically tested for deviating from zero with a one-sample *t*-test (*p* values of <0.0125 were considered significant after Bonferroni correction).

Lastly, per area group, relative frequency distributions were computed for all CF and pRF parameters, with bin sizes of 0.5 deg for CF eccentricity and pRF eccentricity/size or 0.5 mm for CF size. Obtained relative frequency distributions of the controls were averaged, and the 95% CIs of the mean were computed. This allowed us to examine, in addition to their medians, the distributions of the different VFM parameters.

In case relevant differences in CF_RS_ parameters between the patient and the controls were revealed by any of the above comparisons, a similar comparison was made between the CF estimates of the three viewing conditions. In this way, we were able to assess whether the apparent difference between patient and the controls could be potentially attributed to the different viewing instructions they received. For that reason, of primary interest were the comparisons of CF_EO_ vs. CF_EC_ and CF_FIX_ vs. CF_EC_. For each of these comparisons, only those voxels were considered for which the models explained at least 20% of the variance in the time series of both viewing conditions to ensure an evaluation of the same set of voxels. For the CF_EC_, this resulted in two sets of voxels: one for the comparison with CF_EO_ (i.e., CF_EC(EO)_) and one for the comparison with CF_FIX_ (i.e., CF_EC(FIX)_).

To examine the presence of ipsilateral visual field representations in the right hemisphere, a comparison was made between the two bilateral pRF models and the unilateral pRF model by comparing their VE distributions. Here, we limited ourselves to V1, since bilateral representations have been shown before for V1 [11–13] and since late visual areas are already known to sometimes possess large pRFs that overlap with the ipsilateral visual field [16, 21]. Only those voxels were considered for which all models explained at least 20% of the variance in the time series, ensuring evaluation of the same set of voxels for each model. The three models were compared in pairs, with the VE distribution of the first model taken as the test distribution, and the VE distribution of the second model taken as the baseline distribution. By iteratively changing the VE threshold (varying from 0.20 up to 0.99), the hit (HIT) rates (proportion of the test distribution passing the threshold) and false alarm (FA) rates (proportion of the baseline distribution passing the threshold) were computed. For each of the participants, and each of the model comparisons, isosensitivity curves were created by plotting the HIT rates against the FA rates. The area under the curve (AUC) of each of the isosensitivity lines described which model predicted the data best. AUC values above 0.5 indicated that the test model predicted the data better than the baseline model and vice versa. An AUC value of 0.5 indicated that the test and baseline model predicted the data equally well. For each of the model comparisons, a 95% CI was computed by bootstrapping the test and baseline distribution 2000 times with replacement.

## 3. Results

We evaluated the visual field map properties in a hemispherectomy patient, using fMRI, to seek for the presence of functional reorganization of her visual system. More specifically, we evaluated CF properties both when at rest (resting-state (RS)) (CF_RS_) and when visually stimulated (active-state (AS)) (CF_AS_). Median values of the CF_AS_, CF_RS_, and pRF size and eccentricity estimates are presented in Table 1, for the patient and across the controls. These indicate that various differences exist between the patient and the controls.

### 3.1. Connective Field (CF) Eccentricity and Size

#### 3.1.1. Patient vs. Controls

**Figure 2:**
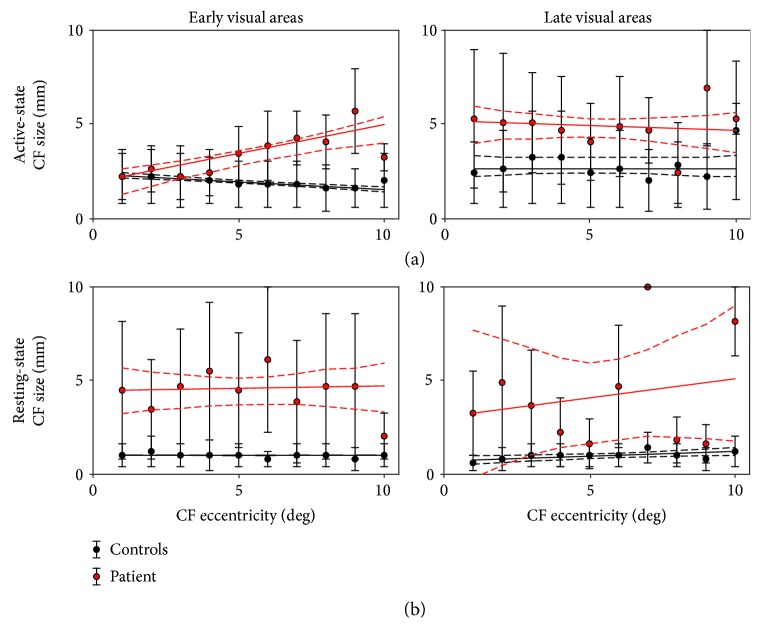
Relationship between eccentricity and size for CF_AS_ (a) and CF_RS_ (b). Data for both area groups of interest, grouped over controls and for the patient (red). CF eccentricity was binned in intervals of 1 deg. Median CF eccentricities are plotted, for which quantile regression fits were calculated (solid red line). Dashed lines represent the 95% bootstrapped confidence intervals for the fit of the median (1000 iterations).

Figure 2(a) shows the relationship between eccentricity and size for CF_AS_ for both area groups. In the early visual areas, the patient shows an increase in CF_AS_ size with the eccentricity bin (*β* = 3.09, *p* = 0.007, df = 8), which is much larger (Δ*β* = 14.52, *F* = 19.01, *p* < 0.001) than that of controls in the controls (*β* = ‐11.43, *p* = 0.008, df = 8). For the late visual areas, we found larger median CF_AS_ overall (mean Δ = 2.00, paired *t*-test: *t* = 4.62, *p* = 0.0013, df = 9). Figure 2(b) shows the relationship between eccentricity and size of CF_RS_. For both area groups, the CF_RS_ of the patient is larger at all eccentricities (paired *t*-test; early visual areas: mean Δ = 3.41, *t* = 9.145, *p* < 0.001, df = 9; late visual areas: mean Δ = 3.20, *t* = 3.73, *p* = 0.0047, df = 9).

**Figure 3:**
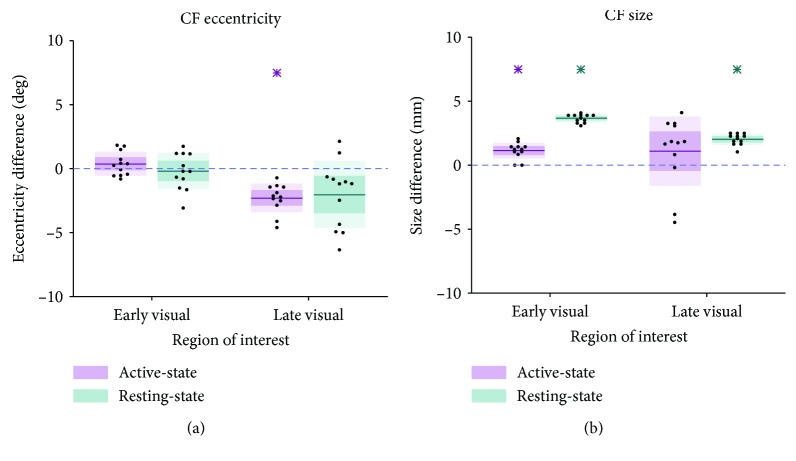
Difference distributions of the medians (patient − control_*n*_). (a) Differences in median CF eccentricity (deg) per area group. (b) Differences in median CF size (in mm) per area group. Pink: CF_AS_ data; blue: CF_RS_ data; thick line: mean difference over all participants, with the dark and lighter shaded areas representing one SD and the 95% confidence interval of the mean, respectively. Blue dashed line at zero indicates no difference between the patient and control. An asterisk indicates a distribution deviating significantly from zero (*p* < 0.0125).

Figure 3 compares, per area group, the difference in median eccentricity (Figure 3(a)) and size (Figure 3(b)) for CF_AS_ (purple) and CF_RS_ (blue) between the patient and the controls. This revealed various differences between the patient and controls. Namely, we found a lower median CF_AS_ eccentricity for the late visual areas (one-sample *t*-test: *t* = ‐7.10, *p* < 0.001, df = 11) and a larger median CF_AS_ for early visual areas (one-sample *t*-test: *t* = 6.23, *p* < 0.001, df = 11). Furthermore, we found larger median CF_RS_ for both early and late visual areas (one-sample *t*-test: *t* = 38.9, *p* < 0.001, and df = 11 and *t* = 16.0, *p* < 0.001, and df = 11, respectively).

**Figure 4:**
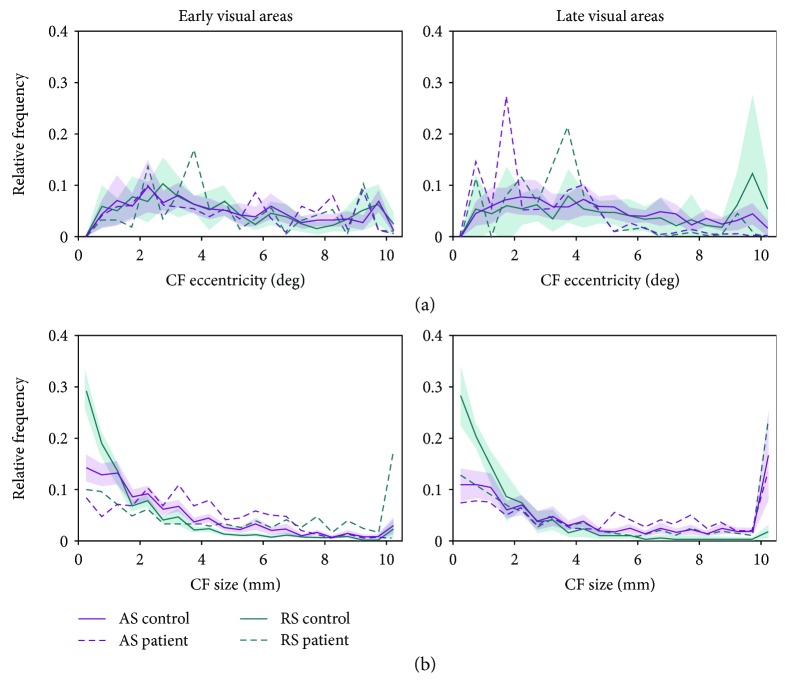
Relative frequency distributions of CF_AS_ (pink) and CF_RS_ (green) eccentricity (a) and size (b), per area group. Solid lines: average across controls (*n* = 12); shaded areas: 95% CI; dashed lines: patient.

Figure 4 shows the associated relative frequencydistributions for CF eccentricity (Figure 4(a)) and size (Figure 4(b)) to illustrate the origin of the differences described above. The distributions for CF_AS_ eccentricity indicate that the patient has a larger proportion of voxels with low eccentricities (range 1-2 deg) in late visual areas. Furthermore, it shows a smaller proportion of voxels with small CF_AS_ (up to 2 mm) in the patient, in early visual areas. This is accompanied by a larger proportion of CF_AS_ in the range of 3-7 mm. For CF_RS_, we found smaller proportions of small CF_RS_ (<2 mm) for both area groups, which are accompanied by larger proportions of large CF_RS_ (>9 mm).

#### 3.1.2. Eyes Closed vs. Fixated and Eyes Closed vs. Open

Our primary finding revealed larger CF_RS_ in the patient compared to the controls, in both early and late visual areas. To assess whether these observations could potentially be attributed to differences in viewing behavior, we compared the CF sizes in three viewing conditions (eyes open (EO), eyes fixated (FIX), and eyes closed (EC)) in four additional control participants.  Table 2 shows the median values of CF_EO_, CF_FIX_, CF_EC(EO)_, and CF_EC(FIX)_. The median CF_EO_ and CF_FIX_ are slightly higher than CF_EC(EO)_ and CF_EC(FIX)_, respectively. Pairwise comparisons of the CF size for the early visual areas revealed no difference between CF_EO_ and CF_EC(EO)_ (paired *t*-test; *t* = ‐0.95, df = 1142, *p* = 0.34) but did reveal a difference between CF_FIX_ and CF_EC(FIX)_ (paired *t*-test; *t* = 2.09, df = 989, *p* = 0.04). Furthermore, we found a difference between CF_EO_ and CF_EC(EO)_ (paired *t*-test; *t* = 4.16, df = 647, *p* < 0.001) and a difference between CF_FIX_ and CF_EC(FIX)_ (paired *t*-test; *t* = 2.61, df = 443, *p* = 0.0095) for the late visual areas. *p* values are uncorrected for spatial autocorrelation and the upsampling that has been applied to the data during the CF modelling.

**Figure 5:**
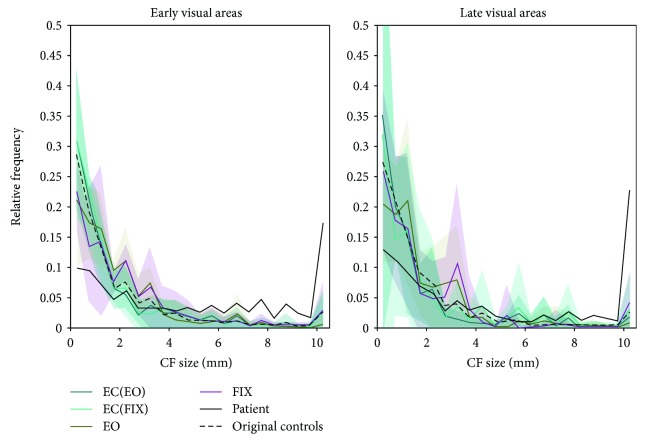
Relative frequency distributions of both CF_EC_ samples (blue lines), CF_EO_ (green), and CF_FIX_ purple size, per area group. Solid colored lines: average across controls (*n* = 4); shaded areas: 95% CI. Additionally, the CF size distribution of the patient (solid black) and the average distribution of the original groups of controls (dashed black, *n* = 12) are plotted.

Figure 5 shows the associated relative frequency distributions of CF sizes for each of the three viewing conditions. This illustrates the origin of the differences in CF size between viewing conditions as described above. In both area groups, the distributions show a slightly larger proportion of voxels with small CFs (2-4 mm) for both the CF_EO_ and CF_FIX_ compared to CF_EC_. In addition, the distribution of the patient (solid black line) and the mean distribution of the original group of controls (dashed black line) are presented that allowed for a direct comparison of all CF size distributions. From this, it can be noted that the origin of the CF size difference for CF_EO_ and CF_FIX_ is different from that of the patient (i.e., she has a smaller proportion of CF sizes in the 0-2 mm range and a larger proportion of CF sizes in the 9-10 mm range).

### 3.2. Population Receptive Field (pRF) Eccentricity and Size

**Figure 6:**
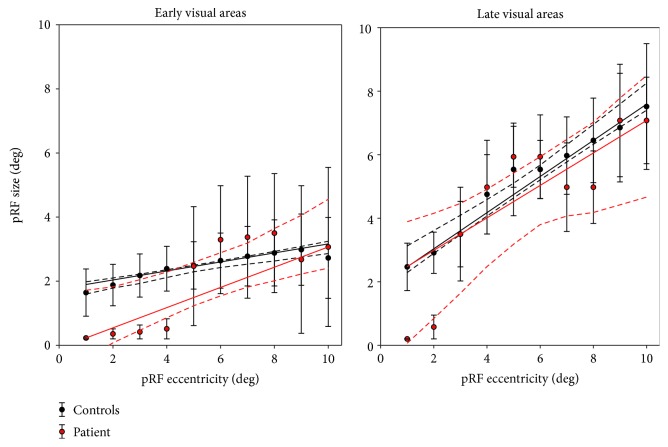
Relationship between pRF eccentricity and size. Data are presented for both area groups of interest, grouped over controls (black) versus the patient (red). PRF eccentricity was binned in intervals of 1 deg. Median pRF eccentricities are plotted, for which quantile regression fits were calculated (solid red line). Dashed lines represent the 95% bootstrapped confidence intervals for the fit of the median (1000 iterations).

Figure 6 shows the relationship between pRF eccentricity and size for both area groups. In the patient, in both early and late visual areas, we found smaller pRFs at the low eccentricities. At higher eccentricities, pRFs are within the normal range. These observations are in line with the previous observations of deviating pRF parameters in this patient [7].

**Figure 7:**
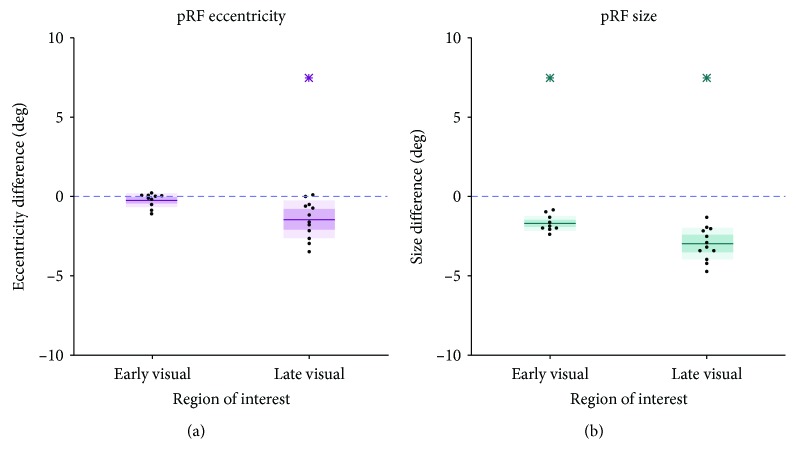
Difference distributions of the medians (patient − control_*n*_). (a) Difference in pRF eccentricity per area group. (b) Same for pRF size. Thick line: mean difference over all participants, the dark and lighter shaded areas representing one SD and the 95% confidence interval of the mean, respectively. Blue dashed line at zero indicates no difference between the patient and control. Asterisk indicates a distribution deviating significantly from zero (*p* < 0.01).

Figure 7 compares the median eccentricity and size in both area groups for the patient and control. In the patient, for the late visual areas, we found a lower median pRF eccentricity. In addition, we found a smaller median pRF size in the early visual cortex and a substantially smaller median pRF size in the late visual areas (all *p* < 0.01).

**Figure 8:**
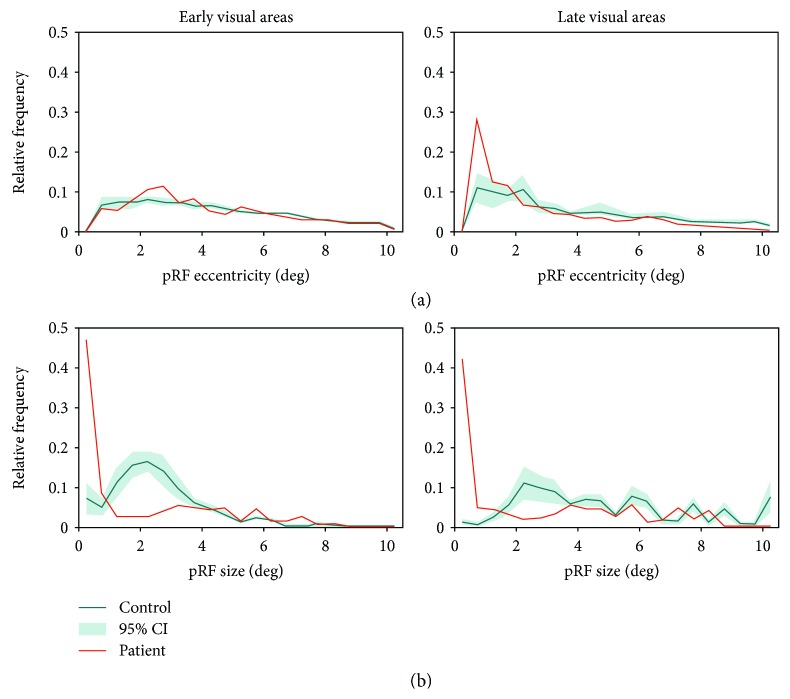
Relative frequency distribution for pRF eccentricity (a) and size (b). Green line: average of controls (*n* = 12); green shaded area: 95% CI; red line: patient.

To enable a more detailed evaluation of the differences between the patient and the control, Figure 8 shows the relative frequency distributions for pRF eccentricity (Figure 8(a)) and size (Figure 8(b)). The distributions for pRF eccentricity indicate that the patient has a larger proportion of voxels with low pRF eccentricities in the late visual areas (at 1 deg). Furthermore, it shows a larger proportion of voxels with small pRF sizes (up to 1 deg) in the patient, in both early and late visual areas. This is accompanied by a smaller proportion of pRFs in the range of 1-3 deg.

### 3.3. Follow-Up Analyses

Previous literature on CFs for V1 established that the sampling from V1 does not vary as a function of eccentricity [8, 22, 23]. To facilitate the interpretation of the (thus unexpected) increase in CF size with eccentricity in the early visual areas of the patient, we separately examined the pRF sizes of the source (i.e., V1) and the target regions of these CFs (i.e., V2 and V3). For the patient, we found a smaller median pRF size in V1 (*p* < 0.01), but not in V2 and V3, compared to controls. A larger CF size (i.e., a higher interareal sampling resolution) in the early visual areas of the patient may have given rise to this change in pRF size (from smaller to normal) from V1 to V2 and V3 that was observed at the lower eccentricities.

### 3.4. Single versus Bilateral pRF Model Comparison

**Figure 9:**
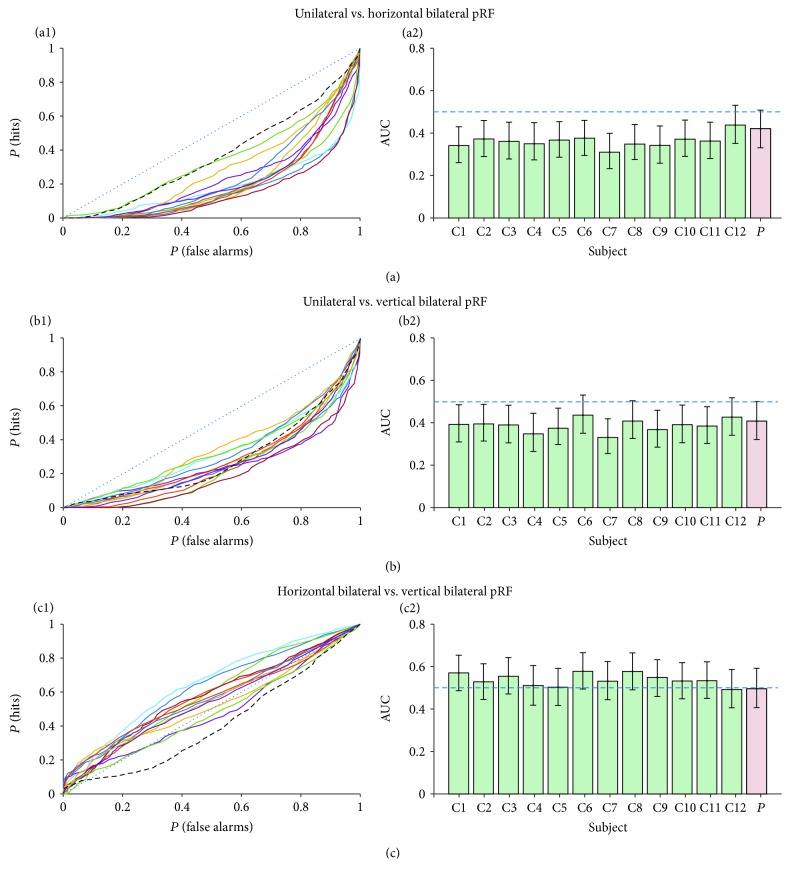
Isosensitivity and AUC plots for model comparison. (a1, b1, c1) Isosensitivity lines for the different model comparisons. Control participants are presented in colored solid lines and the patient in a dashed black line. Bisection line (AUC = 0.5) is plotted in dotted blue. (a2, b2, c2) A bar graph of the AUC values per participant. Error bars represent the 95% CI.

We evaluated a possible ipsilateral contribution to the patient's cortical VFM by applying bilateral pRF modelling. In V1, the three different models (one unilateral and two bilateral) were evaluated using the isosensitivity curves. The isosensitivity lines for the patient showed a similar course as those of the controls, for the two single-versus-bilateral model comparisons (see Figures 9(a1), 9(b1), and 9(c1)). Specifically, they lie below the bisection line (the blue dotted line), suggesting that the single pRF model outperformed both the bilateral models. Bootstrapping analyses of the AUCs showed that the single pRF model performed better than the bilateral models for the majority of the participants (see Figures 9(a2), 9(b2), and 9(c2)), as indicated by the confidence intervals that do not overlap with 0.5. Nevertheless, for none of the participants, the AUC value was significantly larger than 0.5. Thus, by Occam's razor, the unilateral model was adopted as it is the simplest (i.e., with the fewest parameters) and performs at least as well as the more complex model (i.e., with more parameters). The isosensitivity lines of the vertically versus the horizontally mirrored pRF model lie around the bisection line, suggesting that one model did not perform better than the other. This was confirmed by the bootstrapping analyses that showed no AUC values significantly deviating from 0.5.

## 4. Discussion

Our main finding of the evaluation of the VFM properties of a hemispherectomy patient is that both the early and late visual areas contained larger CFs; this is most evident when in a resting-state condition. Additionally, we found smaller pRFs at low eccentricity, primarily for the late visual areas. Lastly, unlike what has been reported for cases with a congenital cause for an absence of a hemisphere, we found no evidence that V1 processes information from two bilateral regions of the visual field. This indicates a strictly contralateral processing of visual spatial information, in line with the patient's perimetrically established homonymous hemianopia.

### 4.1. Enlarged CFs in the Case of a Single Hemisphere

The main advance of this study, compared to our previous report [7], is that we were able to analyze the CF properties in the patient, both for active- and resting-state acquired signals. For the active-state condition, we found an increase in CF size with eccentricity in the early visual areas of the patient. In line with previous findings [7], this increase was absent in the controls. The decreased slope in the patient's later visual areas, compared to her early visual areas, suggests that the nonconstant sampling in these areas is largely inherited from V2/V3. For the resting-state condition, we also found larger CFs, this time in both area groups and over practically the entire range of eccentricities.

To our knowledge, our study is the first to find abnormally large CFs in a clinical case to date. Three other studies have examined CF parameters in a clinical population with a visual deficit. In patients with macular degeneration, Haak et al. showed preserved corticocortical organization, when visually stimulated, between input-deprived portions of V1 > V2/V3 [24]. In early blind and anophthalmia cases, Bock et al. found an intact corticocortical organization between V1 > V2/V3, even despite a complete absence of visual experience and retinal input [25]. Recently, Ahmadi et al. [26] showed changes in corticocortical connections for V1 > V3 in albinotic participants. Hence, this raises the question whether the abnormally large CF in our case is a sign of functional reorganization. While the absence of one hemisphere and half of the visual field might be a strong incentive for reorganization to occur [11, 12], it may also change the processing in the remaining hemisphere without reorganization per se. Therefore, before interpreting the observed differences in terms of functional reorganization, we should be able to rule out other possible causes [27].

Rather than the other two studies, ours presents the CF estimates in the case of a single hemisphere in the absence of the other one. What might explain the enlarged CFs in our case? And how can the enlarged CFs be reconciled with the observed pRF sizes, which were smaller than normal in foveal regions and roughly normal in the visual periphery? Indeed, it seems reasonable to expect that the pRF size scales with the CF size because larger CFs cover a larger cortical area and therefore cover a wider range of visual field locations. For example, if a voxel in V3 samples from a large area of V1 (i.e., it has a large CF), it intrinsically represents a large area of visual field. However, unlike the stimulus-referred pRF, the neural-referred CF captures both excitatory and suppressive responses [8]. Receptive fields can be conceptualized as spatial tuning curves with an excitatory center and a suppressive surround, the latter of which is thought to consist of a classical surround underpinned by lateral (intra-areal) connections and a wider extraclassical surround linked to feedback connections from higher order visual areas [8, 26–29]. The single Gaussian pRF model used in the present work captures only the excitatory responses and therefore reflects the center of the spatial tuning curve [27]. Since the pRF was not enlarged in the patient, and given that the CFs model interareal connectivity, we speculate that the enlarged CFs reflect a change in the extraclassical suppressive surround underpinned by modulatory feedback connections from later visual areas. This account suggests that the absence of the opposing cerebral hemisphere perturbs the feedback connectivity between V1 and later visual areas, the function of which is normally highly dependent on interhemispheric interactions (e.g., they have large receptive fields that overlap with the vertical meridian of the visual field). The account is also consistent with the observation that the CF enlargement is most profound in the absence of visual stimulation. When visually stimulated, the brain's activity is largely dominated by feed forward processes [29]. This makes the neural-referred receptive fields, estimated when visually active, less susceptible to feedback perturbations. This is in contrast with their estimations at rest and thus based on intrinsic brain activity. Whether these perturbations are the result of plastic reorganization or a consequence of unmasked suppressive feedback signals that are normally masked by interhemispheric signaling is an open question that remains to be addressed in future work.

However, the larger CFs in the early visual areas may also be a response to the smaller pRFs found in the early visual areas. In this case, their increased size may compensate for a lack of signal integration at this earlier level of processing in the cortical hierarchy. Indeed, detailed inspection of the pRF sizes in the source (i.e., V1) and target regions (i.e., V2 and V3) of these CF estimations revealed smaller pRFs in the patient, exclusive to V1. While one would expect that these smaller V1 pRFs would be carried over to later visual areas, this is not what we found. A possible explanation is that the relatively large CFs of the patient for early visual areas V2 and V3 compensate for the small pRFs in V1. In this way, the pRF sizes of later stages of visual processing would be normalized.

### 4.2. CF Differences during Resting-State Cannot Be Attributed to Differences in Viewing Behavior

To investigate whether the observed increase in CF_RS_ size for the patient might be explained by a difference in viewing behavior, we compared the estimates from RS data acquired under three different viewing conditions (EC, FIX, and EO). Of primary interest were the comparisons of the eyes-open and eyes-fixated (most closely resembling the patient's condition) to the eyes-closed (identical to the controls) condition. Differences between these conditions could affect the interpretation of the enlarged CF_RS_ as found in the patient.

For both the eyes-open and the eyes-fixated condition, we found a slightly larger median CF compared to the eyes-closed condition, in both area groups. Note that since uncorrected statistics have been reported, this difference found is most likely an overestimation of the actual difference. Nevertheless, these differences were much smaller (i.e., ~0.5 mm) than the difference we observed when comparing the patient to the controls (i.e., 3.20 mm). Furthermore, from the relative frequency distribution of CF size, we can conclude that the slight “viewing-condition-dependent” increase in CF size originated from a larger proportion of 2-4 mm range CFs. This is very different from the patient, who had primarily smaller proportions of small range CFs (<2 mm) and larger proportions of large range CFs (>9 mm), compared to controls.

To conclude, the differences in CF observed between the patient and the controls are much larger than can be explained by differences in viewing behavior and also occur in different parts of the CF size spectrum. Therefore, we conclude that the enlarged CFs of the patient are genuine.

### 4.3. Enlarged and More Detailed Foveal Processing

Our pRF modelling revealed a larger proportion of voxels with a low pRF eccentricity (foveal—parafoveal) for the late visual areas. Furthermore, we found larger proportions of small pRFs for both the early and late visual areas. These smaller than normal pRFs were found at a low eccentricity and are hence associated with a (para)foveal vision. These findings are in line with, and complement, previous observations on this patient of an enlarged foveal representation and smaller pRFs in the lateral occipital cortex as compared to controls [7]. This difference in pRF cannot be attributed to poor fixation as the patient was able fixate well. Furthermore, poor fixation would have resulted in larger pRF estimates, whereas in the patient, we found smaller pRF estimates.

Deviating pRF properties have been reported in a number of studies in patients with homonymous hemianopia. In another hemispherectomy patient, compared to controls, larger pRFs and increased eccentricity for dorsal V2 and V3 were found [30]. In a subset of hemianopic patients, Papanikolaou et al. [31] found a slight increase in pRF size in V1 of the intact hemisphere. This contrasts our finding of smaller pRFs at lower and approximately normal-sized pRFs at higher eccentricities in the early visual areas. All studies reported that polar angle representations remained unchanged [7, 29, 30].

Our pRF data implies that in the late visual areas, the patient shows an enlarged foveal representation (as reflected by the larger proportions of low pRF eccentricities), with a detailed processing of the visual field (as reflected by smaller pRFs). Similarly, the patient's early visual areas also possess a more detailed processing of spatial information (as reflected by the smaller pRFs). These findings might be explained as a functional response to the homonymous hemianopia (which was without macular sparing). Patients with central vision loss—for example, due to macular degeneration—often adopt an eccentric preferred retinal locus (PRL; see [31, 32]), which they use as a kind of pseudofovea. We speculate that our patient might have developed such a PRL as well and, in response to that, also a more extended (para)foveal processing in certain brain areas. Unfortunately, at present, we have no means to clinically verify this interpretation.

Haak et al. [7] attributed the unusually small pRFs in the patient's late visual cortex to a lack of input from the opposite cerebral hemisphere. At the same time, both Georgy et al. [30] and Papanikolaou et al. [31] attributed the increase in pRF size in the early visual cortex in their patients to a loss of interhemispheric input as well. Hence, there is no indication that the condition of hemianopia leads to consistent deviations in either field map representations or pRF properties. Differences in cause and onset of the hemianopia may be factors that affect what type of change will occur. At the same time, we should note that the number of cases studied is still very small, which prevents us from drawing firmer conclusions.

### 4.4. No Evidence for Representations of the Ipsilateral Visual Field

We also examined whether the V1 in the remaining hemisphere represented the ipsilateral visual field. Comparison of the three different pRF-mapping models showed no better model fit for the bilateral pRF models compared to the single pRF model, neither for the controls nor the patient. This suggests that the V1 of the patient, like the controls, primarily represented the contralateral visual field. This finding is consistent with the patient's perimetric results (i.e., a homonymous hemianopia).

Despite being described as infrequent, cases of bilateral visual field representations have been reported before in the literature, with patients possessing bilateral visual field representations despite having only one (intact) hemisphere [12] or two underdeveloped hemispheres [33], suggesting functional reorganization of the visual system. More recently, another case of congenital unilateral loss of the cerebral cortex (hemihydranencephaly) with a preserved visual field was investigated [11]. In particular, using the same bilaterally pRF-modelling approach as presented here, they tested for the presence of both ipsilateral and contralateral visual field representations in this patient. Data revealed interleaved representations of both the ipsilateral and the contralateral visual hemifield located in the early visual cortex of the intact hemisphere.

These studies show that in cases of major cortical damage to the visual system, there is a potential for functional reorganization that supports an improved perceptual performance. Yet, no bilateral processing was found in our hemispherectomy patient. It must be noted that most of the cases described above concern congenitally hemiblind observers whereas our case has an acquired cortical hemiblindness. An explanation for this discrepancy could thus be that in the congenitally unihemispheric patient, the abnormal functional organization could be attributed to the fact that there is an absence of the molecular gradient directing the growth of white matter fibers that normally cross the corpus callosum. Without this gradient and with no hemisphere to grow to, white matter fibers remain in the ipsilateral hemisphere allowing for ipsilateral projections. In the case of a hemispherectomy, however, such fibers have already been grown and cut away during surgery. Additionally, molecular gradients are no longer present after the critical period (the present case had her hemispherectomy at the age of three).

## 5. Conclusion

The case presented here provides signs of relatively subtle functional reorganization. The patient's visual system did not recover from her acquired visual hemifield defect that emerged after removal of the left hemisphere. On the other hand, despite the initial major impact of the hemispheric removal on her motoric and linguistic abilities, the girl partially recovered her motor control and speaks bilingually. These aspects do indicate that functional reorganization has taken place after the surgery but not so obviously in the visual domain. In line with this, evaluation of the patient's visual field maps indeed did not reveal any major remapping, in that the remaining hemisphere does not contain representations of the ipsilateral visual field. Hence, we conclude that no large-scale reorganization has taken place.

At the same time, the chronic visual field defect allowed us to examine the effects of an interrupted processing of half of the retinal output and deprivation of interhemispheric inputs on the VFM map properties in the remaining hemisphere. This led to the observation of abnormal VFM properties in both the early and late visual areas. Specifically, we found larger than normal CFs for the patient. These larger CFs may be considered a form of subtle functional reorganization. The more detailed spatial processing and, in the late visual areas, the additional enlarged foveal representation found in the patient could be interpreted as a functional reorganization as well. In particular, in the context of a possible PRL, the brain might have adaptively reorganized itself in response to the development of such eccentric fixation. However, the interpretation of these deviating properties should be done with caution. Often other explanations are able to account for the abnormal maps that do not require assuming cortical reorganization. For example, abnormal visual maps may be reflections of (partially) absent visual inputs [7].

To conclude, in the absence of large-scale functional reorganization, we do find indications for relatively subtle changes in VFM organization, which might facilitate adaptive processing and viewing strategies. This evaluation contributes to the understanding of the consequences of removing a hemisphere at an early developmental stage for the functional organization of the visual system.

## Figures and Tables

**Table 1:** Median eccentricity and size estimates for CF_AS_ and CF_RS_ per area group, for the patient and averaged across controls. The medians' 1^st^ and 3^rd^ quartiles are presented between brackets.

	CF_AS_ ecc	CF_AS_ size	CF_RS_ ecc	CF_RS_ size	pRF ecc	pRF size
Early visual areas						
Patient	4.73 (2.4–7.5)	3.27 (1.8–5.1)	3.96 (2.9–7.2)	4.69 (1.4–8.6)	3.63 (2.3–5.8)	0.61 (0.2–3.6)
Controls	4.28 (2.4–6.9)	2.04 (0.8–3.9)	4.04 (2.4–6.8)	1.02 (0.4–2.4)	3.89 (2.2–6.2)	2.26 (1.5–3.2)

Late visual areas						
Patient	2.11 (1.6–4.1)	5.10 (1.6–8.0)	3.36 (2.2–4.0)	3.06 (1.0–9.2)	1.90 (0.9–4.2)	1.36 (0.2–5.0)
Controls	4.38 (2.5–6.7)	2.86 (1.0–7.3)	4.83 (2.8–8.0)	1.02 (0.4–2.2)	3.36 (1.7–6.1)	4.30 (2.7–6.5)

**Table 2:** Median CF size estimates across subjects of the selected voxels per viewing condition. Interquartile ranges are presented in brackets.

Condition	Early visual	Late visual
CF_FIX_	1.43 (0.61–2.65) (*n* = 990)	1.22 (0.61–2.65) (*n* = 444)

CF_EC(FIX)_	0.82 (0.41–2.25) (*n* = 990)	0.82 (0.20–2.040) (*n* = 444)

CF_EO_	1.22 (0.61–2.45) (*n* = 1143)	1.22 (0.82–2.65) (*n* = 648)

CF_EC(EO)_	1.02 (0.41–2.60) (*n* = 1143)	0.61 (0.20–1.43) (*n* = 648)

## Data Availability

The data used to support the findings of this study are available from the corresponding author upon request.

## References

[1] Werth R. (2006). Visual functions without the occipital lobe or after cerebral hemispherectomy in infancy. *European Journal of Neuroscience*.

[2] Huxlin K. R. (2008). Perceptual plasticity in damaged adult visual systems. *Vision Research*.

[3] Wandell B. A., Smirnakis S. M. (2009). Plasticity and stability of visual field maps in adult primary visual cortex. *Nature Reviews Neuroscience*.

[4] Guzzetta A., D’Acunto G., Rose S., Tinelli F., Boyd R., Cioni G. (2010). Plasticity of the visual system after early brain damage. *Developmental Medicine & Child Neurology*.

[5] Gilbert C. D., Li W. (2012). Adult visual cortical plasticity. *Neuron*.

[6] Reitsma D. C., Mathis J., Ulmer J. L., Mueller W., Maciejewski M. J., DeYoe E. A. (2013). Atypical retinotopic organization of visual cortex in patients with central brain damage: congenital and adult onset. *The Journal of Neuroscience*.

[7] Haak K. V., Langers D. R. M., Renken R., van Dijk P., Borgstein J., Cornelissen F. W. (2014). Abnormal visual field maps in human cortex: a mini-review and a case report. *Cortex*.

[8] Haak K. V., Winawer J., Harvey B. M. (2013). Connective field modeling. *NeuroImage*.

[9] Gravel N., Harvey B., Nordhjem B. (2014). Cortical connective field estimates from resting state FMRI activity. *Frontiers in Neuroscience*.

[10] Carvalho Studying cortical plasticity in ophthalmic and neurological disorders: from stimulus-driven to cortical circuitry approaches.

[11] Fracasso A., Koenraads Y., Porro G. L., Dumoulin S. O. (2016). Bilateral population receptive fields in congenital hemihydranencephaly. *Ophthalmic & Physiological Optics*.

[12] Muckli L., Naumer M. J., Singer W. (2009). Bilateral visual field maps in a patient with only one hemisphere. *Proceedings of the National Academy of Sciences of the United States of America*.

[13] Henriksson L., Raninen A., Nasanen R., Hyvarinen L., Vanni S. (2007). Training-induced cortical representation of a hemianopic hemifield. *Journal of Neurology, Neurosurgery & Psychiatry*.

[14] Gravel N. (2019). *The Neuroanatomical Organization of Intrinsic Brain Activity Measured by FMRI Activity in the Human Visual Cortex, Gravel Araneda, Nicolas Gaspar, [Ph.D. thesis]*.

[15] Pruim R. H. R., Mennes M., van Rooij D., Llera A., Buitelaar J. K., Beckmann C. F. (2015). ICA-AROMA: a robust ICA-based strategy for removing motion artifacts from fMRI data. *NeuroImage*.

[16] Nestares O., Heeger D. J. (2000). Robust multiresolution alignment of MRI brain volumes. *Magnetic Resonance in Medicine*.

[17] Dumoulin S. O., Wandell B. A. (2008). Population receptive field estimates in human visual cortex. *NeuroImage*.

[18] Engel S. A., Glover G. H., Wandell B. A. (1997). Retinotopic organization in human visual cortex and the spatial precision of functional MRI. *Cerebral Cortex*.

[19] Wandell B. A., Dumoulin S. O., Brewer A. A. (2007). Visual field maps in human cortex. *Neuron*.

[20] Sereno M. I., Mcdonald C. T., Allman J. M. Analysis of retinotopic maps in extrastriate cortex. https://www.cogsci.ucsd.edu/~sereno/107B-201/readings/04.07-analysisretinmap.pdf.

[21] Hoffmann M. B., Kaule F. R., Levin N. (2012). Plasticity and stability of the visual system in human achiasma. *Neuron*.

[22] Tootell R. B. H., Mendola J. D., Hadjikhani N. K., Liu A. K., Dale A. M. (1998). The representation of the ipsilateral visual field in human cerebral cortex. *Proceedings of the National Academy of Sciences of the United States of America*.

[23] Harvey B. M., Dumoulin S. O. (2011). The relationship between cortical magnification factor and population receptive field size in human visual cortex: constancies in cortical architecture. *The Journal of Neuroscience*.

[24] Bock A. S., Binda P., Benson N. C., Bridge H., Watkins K. E., Fine I. (2015). Resting-state retinotopic organization in the absence of retinal input and visual experience. *The Journal of Neuroscience*.

[25] Ahmadi K., Herbik A., Wagner M., Kanowski M., Thieme H., Hoffmann M. B. (2019). Population receptive field and connectivity properties of the early visual cortex in human albinism. *bioRxiv*.

[26] Haak K. V., Cornelissen F. W., Morland A. B. (2012). Population receptive field dynamics in human visual cortex. *PLoS One*.

[27] Schwabe L., Ichida J. M., Shushruth S., Mangapathy P., Angelucci A. (2010). Contrast-dependence of surround suppression in macaque V1: experimental testing of a recurrent network model. *NeuroImage*.

[28] Schwabe L., Obermayer K., Angelucci A., Bressloff P. C. (2006). The role of feedback in shaping the extra-classical receptive field of cortical neurons: a recurrent network model. *The Journal of Neuroscience*.

[29] Lamme V. A. F., Roelfsema P. R. (2000). The distinct modes of vision offered by feedforward and recurrent processing. *Trends in Neurosciences*.

[30] Georgy L., Jans B., Tamietto M., Ptito A. (2019). Functional reorganization of population receptive fields in a Hemispherectomy patient with blindsight. *Neuropsychologia*.

[31] Papanikolaou A., Keliris G. A., Papageorgiou T. D. (2014). Population receptive field analysis of the primary visual cortex complements perimetry in patients with homonymous visual field defects. *Proceedings of the National Academy of Sciences of the United States of America*.

[32] Von Noorden G. K., Mackensen G. (1962). Phenomenology of eccentric fixation. *American Journal of Ophthalmology*.

[33] Ptito M., Dalby M., Gjedde A. (1999). Visual field recovery in a patient with bilateral occipital lobe damage. *Acta Neurologica Scandinavica*.

